# Human Alu loci gain enhancer-type epigenetic modifications in monocytes during response to bacterial LPS

**DOI:** 10.1186/s13072-026-00686-x

**Published:** 2026-07-10

**Authors:** Yonghao Pan, Kenji Ichiyanagi

**Affiliations:** Laboratory of Genome and Epigenome Dynamics, Department of Animal Sciences, Graduate School of Bioagricultural Sciences, Nagoya University, Furo-cho, Chikusa- ku, Nagoya, 464-8601 Japan

**Keywords:** Alu element, Enhancer regulation, Innate immunity, Epigenomics

## Abstract

**Background:**

Bacterial lipopolysaccharide (LPS) potently activates innate immunity. Transposable elements (TEs), particularly lineage-specific short interspersed elements (SINEs), have been implicated in immune regulatory evolution, yet their epigenomic roles during immune activation remain unclear.

**Results:**

Here, we reanalyzed published ChIP-seq data for H3K4me1, H3K4me3, and H3K27ac from human monocytes and mouse macrophage-like cells under basal and LPS-stimulated conditions. We identified widespread and dynamic activation of Alu-associated regulatory regions in human monocytes, accounting for around 15% of total enhancer-associated ChIP-seq peaks with and without LPS stimulation. In particular, over one quarter of the Alu-containing H3K4me1 and H3K27ac peaks appeared only after LPS stimulation. The Alu-containing enhancers that appeared in short treatment with LPS linked to many genes involved in the acute immune and inflammatory responses. On the other hand, prolonged LPS stimulation produced stronger chromatin activation at Alu loci that were linked to genes with more dispersed pathway associations, likely reflecting post-stimulation adaptation or tolerance.

**Conclusions:**

These findings suggest that Alu elements act as regulatory scaffolds that shape the temporal and functional landscape of human immune responses. Our findings suggest that Alu elements, as primate-specific TEs, may contribute to heightened sensitivity to LPS by shaping species-specific enhancer landscapes.

**Supplementary Information:**

The online version contains supplementary material available at https://doi.org/10.1186/s13072-026-00686-x.

## Introduction

Mammalian cells recognize bacterial lipopolysaccharide (LPS) as a pathogen-associated molecular pattern to initiate immune responses. The receptors, such as Toll-like receptor 4 (TLR4) [1], cluster of differentiation 14 (CD14) [2], responsible for mediating LPS recognition, are relatively conserved among species. However, the sensitivity to LPS is highly variable among species [3, 4]. Humans generally exhibit greater sensitivity to LPS stimulation than mice, rats, rabbits, pigs, cows, sheep, and baboons under many experimental conditions. Among the immune cells, the monocyte–macrophage lineage plays a central role for responding to LPS [5]. Previous studies have extensively characterized the transcriptional and epigenomic landscapes induced by LPS stimulation [6–8]. However, relatively little is known about the mechanisms underlying the regulation of epigenome dynamics.

Transposable elements (TEs) have increasingly been recognized as important contributors to the evolution of mammalian immune regulation. TEs constitute approximately 45% of the human genome [9] and 37.5% of the mouse genome [10], and many have been co-opted as regulatory elements in innate immunity [11, 12]. Short interspersed elements (SINEs) account for over 10% of both genomes, with Alu elements predominating in humans and B1 and B2 in mice [13]. Alu represents the most abundant primate-specific SINE family with over one million copies residing in the human genome, and constitutes a substantial fraction of potential regulatory substrates. Alu is derived from 7SL RNA and typically consists of two monomeric units, called left and right arms, respectively, separated by an A-rich linker. Rodent B1 is also derived from 7SL RNA, but it is comprised by a monomer that corresponds to the Alu left arm carrying an RNA Pol III promoter. On the other hand, rodent B2 is derived from tRNA, and thus the B2 sequences are dissimilar to those of Alu and B1 [14]. Alu elements are classified into three major subfamilies—the oldest AluJ (showing the highest levels of sequence divergence), intermediate AluS, and the youngest AluY (showing the lowest divergence)—reflecting successive waves of retrotranspositional activity during primate evolution [15]. B1 and B2 have likewise undergone lineage-specific expansions in rodents, resulting in distinct SINE repertoires residing at non-orthologous loci between primates and rodents. These evolutionary differences raise the possibility that SINE families contribute to species-specific regulatory landscapes [16].

Previous studies have shown that Alu elements can contribute to enhancer function by providing transcription factor binding motifs and responding to diverse cellular stimuli [17–19]. Multiple transcription factors involved in immune responses, stress signaling, and development have been reported to bind Alu sequences, suggesting a potential role of Alu elements as reservoirs of regulatory motifs. Based on these observations, we hypothesized that Alu elements may participate in the epigenomic response to LPS stimulation in human monocytes. Specifically, we sought to determine whether Alu-associated regulatory regions play an active role in LPS-induced enhancer activation across different regulatory scales, or whether their apparent activation merely reflects global changes in enhancer strength during stimulation. To address this question, we systematically analyzed publicly available epigenomic datasets from monocytes under LPS stimulation.

## Methods

### mRNA-seq processing

The mRNA-seq datasets were obtained from Short Read Archive (SRA) (accession, GSE85243, additional file 1: Table S1). The datasets were single-end reads of 42 bp in length. Raw sequencing adaptors and low-quality bases were trimmed using Trim Galore！ [20] with parameters appropriate for single-end libraries. Clean reads were aligned to the mouse (mm10) or human (hg38) reference genomes using STAR (https://github.com/alexdobin/STAR) using --outFilterMultimapNmax 100 and subsequently sorted with SAMtools [21]. The raw counts were measured by featureCounts [22].

### ChIP-seq processing and peak calling

The ChIP-seq dataset were obtained from SRA (human monocytes, histone ChIP, GSE85245; mouse macrophage-like cells, histone ChIP, GSE91007; human iPSC-derived microglia, MEF2C ChIP, GSE306993) (additional file 1: Table S2). The H3K4me1, H3K4me3, and H3K27ac ChIP-seq libraries were prepared from the same cells at each time point with two and one replicates for human and mouse, respectively. The datasets were single-end reads from human and mouse. The read lengths were 75 and 51 bp for human and mouse samples, respectively. Raw sequencing adaptors and low-quality bases were trimmed using Trim Galore！ [20] with parameters appropriate for single-end libraries. Clean reads were aligned to the mouse (mm10) or human (hg38) reference genomes using Bowtie2 [23] and subsequently sorted and filtered with q30 with SAMtools [21] to remove reads mapped to multiple regions. PCR duplicates were removed using Picard (http://broadinstitute.github.io/picard/). Peak calling was performed with MACS2 [24], using -f BAM -q 0.01 for narrow marks (H3K4me3, H3K27ac, and transcription factors) and -f BAM --broad --broad-cutoff 0.01 for broad marks (H3K4me1). Peak sets were merged and intersected using bedtools [25]. BAM files were indexed with SAMtools, and normalized signal tracks were generated using deepTools [26] with the following parameters: --binSize 10 --normalizeUsing CPM --extendReads 200 --centerReads --smoothLength 60 -p max. Peak signals were transformed by t-scaling before applying k-means clustering by R.

### Identification of super-enhancers

ROSE algorithm [27] was employed to identify SEs. The analyses were conducted on BAM files derived from H3K27ac ChIP-seq data from human monocytes with and without LPS stimulation. The total peaks identified in the respective conditions were subjected to ROSE analysis.

### Enhancer definitions

Chromatin states representing enhancers and promoters were inferred using ChromHMM [28]. ChIP-seq BAM files for H3K4me1, H3K4me3, and H3K27ac were binarized with the BinarizeBam function (hg38 genome size). A full-stack model comprising 8 chromatin states was learned using the LearnModel module with a 200 bp resolution. Based on emission probabilities, States 6 (with strong H3K4me1, strong H3K27ac, and no H3K4me3) was classified as active enhancers.

### Analysis of sequence motifs in enhancers

The motif annotation of the candidate enhancers was carried out by using HOMER [29] with the function of findMotifsGenome.pl with -size given parameters. To identify the location of MEF2 family motifs, the consensus sequences of representative Alu subfamilies were analyzed by FIMO [30].

### Hi-C data processing

The Hi-C datasets were downloaded from SRA (GSE201353, additional file 1; Table S3). Hi-C sequencing reads were mapped to the human reference genome (hg38) using Chromap [31]. PCR duplicates were removed using Picard. Valid paired reads were extracted and processed with pairtools [32]. Contact matrices were generated in .cool format at 5-kb resolution using cooltools [33]. The resulting contact matrices were balanced using the balance function implemented in cooler [34]. For downstream analyses, cooltools was used to compute genome-wide expected contact frequencies (compute-expected), identify A/B compartments (call-compartments), and detect insulation profiles (diamond-insulation). Topologically associating domains (TADs) were identified using the cooltools insulation function with window sizes of 200 kb, 400 kb, and 800 kb, applying the Li threshold for boundary detection.

### Enhancer-associated genes identified

The candidate genes that could be under the control of a given enhancer were first identified with GREAT [35] in the settings: Basal+extension: 5 kb upstream, 1 kb downstream, and 100 kb max extension in hg38. The candidate gene lists were filtered by the Hi-C data; only the genes and enhancers in the same TAD were retained, thereby restricting enhancer–gene associations to a biologically relevant 3D chromatin neighborhood and reducing spurious long-range assignments. Finally, only expressed genes showing 10 or more mapped reads in the respective mRNA-seq data were regarded as enhancer-associated transcribed genes.

### Signaling pathway enrichment

Integrated pathway analysis was performed using Kyoto Encyclopedia of Genes and Genomes (KEGG) and gene ontology (GO) pathways in clusterProfiler [36] with enrichGO and enrichKEGG functions, the redundancy was removed by the simplify function.

## Results

### LPS induces distinct epigenomic landscapes at Alu sites in human monocytes

**Fig. 1:**
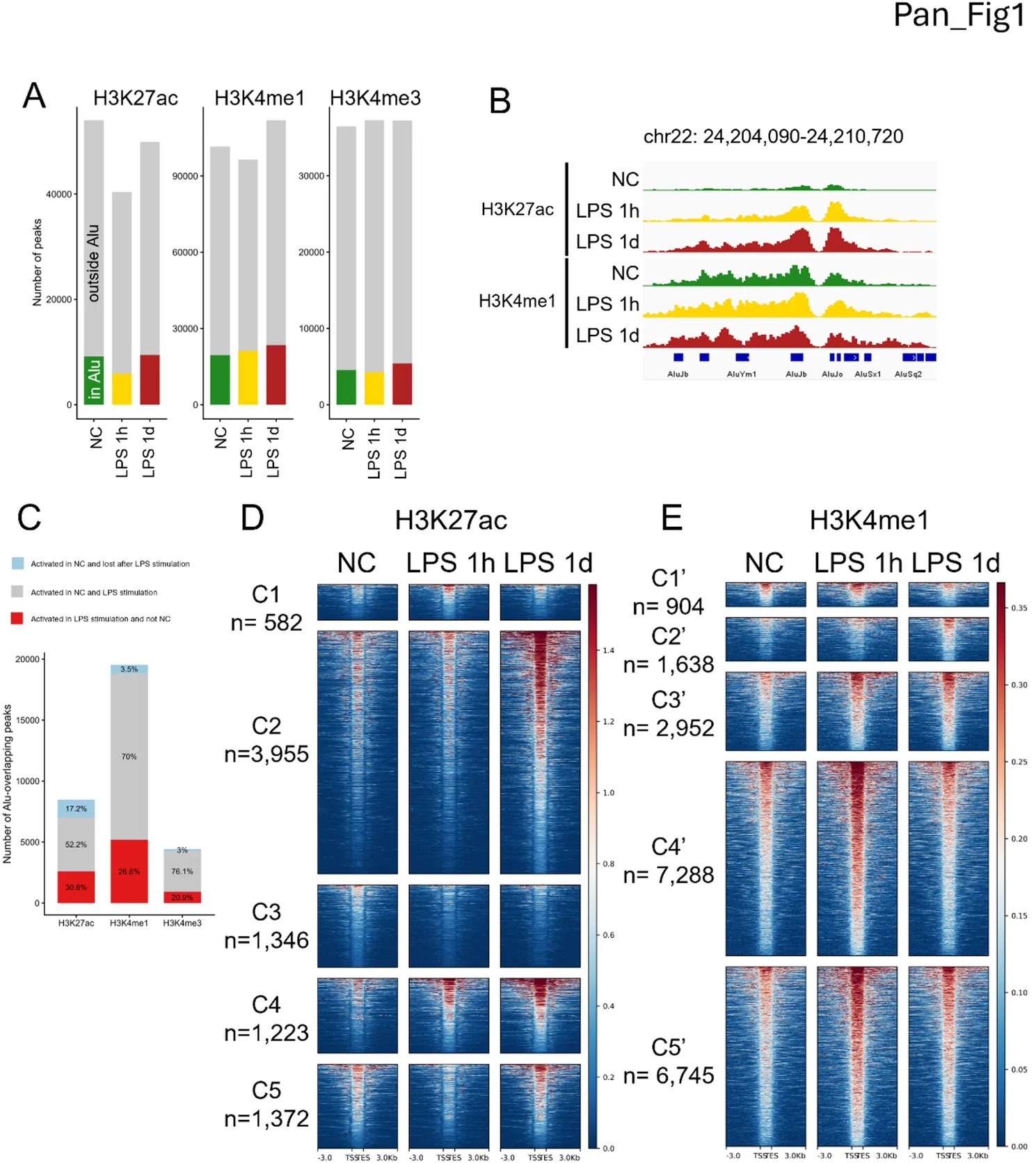
Alu-containing ChIP-seq peaks under the three conditions. **A** Numbers and proportions of Alu-containing peaks relative to total peaks of H3K27ac, H3K4me1, and H3K4me3 under the three conditions, NC, LPS 1 h, and LPS 1 d. Peaks of which overlapped were located within Alu and are colored in green (NC), yellow (LPS 1 h), and red (LPS 1d). **B** Representative IGV tracks showing H3K27ac and H3K4me1 signals around the peaks of which regions were located within Alu. **C** Proportions of Alu-contained peaks across NC and LPS stimulation conditions **D**–**E** Heat map representation of H3K27ac **D** and H3K4me1 **E** signals. The Alu-containing peaks were categorized by k-means clustering

To identify the epigenetic states of the SINE sites under the stimulation with LPS, we analyzed the published chromatin immunoprecipitation sequencing (ChIP-seq) data for active histone modifications, H3K4me1, H3K4me3, and H3K27ac, from human monocytes. We first identified genome-wide ChIP-seq peaks under three conditions: negative control (NC), stimulated by LPS for an hour (LPS 1 h), and stimulated by LPS for a day (LPS 1 d). Approximately 40,000 H3K27ac peaks, 90,000 H3K4me1 peaks, and 35,000 H3K4me3 peaks were detected per condition. Among these, ~ 15% of peaks overlapped with Alu sequences (Fig. 1A and B). Notably, more than one quarter of the Alu-containing H3K27ac and H3K4me1peaks appeared only after LPS treatment. (Fig. 1C).

To characterize the epigenetic dynamics of these regulatory regions, we applied k-means clustering (k = 5) to Alu-containing H3K27ac and H3K4me1 peaks based on their signal profiles across the three conditions. For H3K27ac, 8,478 Alu-containing peaks were classified into five distinct clusters (Fig. 1D). These included: LPS-rapid response peaks showing maximal signal at LPS 1 h (n = 582, 6.9%, C1); LPS-late response peaks with highest signal at LPS 1 d (n = 3,955, 47%, C2); LPS-repressed peaks with strongest signal under NC conditions (n = 1,346, 16%, C3); LPS-upregulated peaks exhibiting elevated signal under both LPS conditions (n = 1,223, 14%, C4); and LPS-rapid repression peaks showing transient signal loss at LPS 1 h followed by recovery at LPS 1 d (n = 1,372, 16%, C5). Notably, the majority of Alu-containing H3K27ac peaks (n = 5,760; 68%) displayed increased signal following LPS stimulation, with the strongest average enrichment observed at the time point of LPS 1d, indicating a predominant association of Alu elements with inducible enhancer activation. The same clustering analysis for Alu-containing H3K4me1 peaks (Fig. 1D) categorized the 19,527 peaks into five groups: LPS-late repression (n = 904, 4.6%, C1’), LPS-late activation (*n* = 1,638, 8.3%, C2’), LPS-upregulated (*n* = 2,952, 16%, C3’), LPS-rapid response (*n* = 7,288, 37%, C4’), and LPS-recovery clusters (*n* = 6,745, 35%, C5’), reflecting diverse but largely LPS-increased H3K4me1 dynamics. Collectively, more than two-thirds of Alu-containing H3K27ac peaks exhibited LPS-induced activation, and the vast majority of Alu-containing H3K4me1 peaks showed higher signal levels under LPS-stimulated conditions compared with NC, supporting a widespread involvement of Alu-associated regions in LPS-responsive enhancer landscapes (Fig. 1D and E).

The involvement of Alu in LPS-induced epigenome changes prompted us to analyze whether the mouse SINEs are also involved in the LPS response. To this end, we analyzed H3K27ac and H3K4me1 ChIP-seq data for mouse monocyte-like cells under three conditions: negative control (NC), stimulated by LPS for an hour (LPS 1 h), and stimulated by LPS for four hours (LPS 4 h). Peaks containing B1 and B2 elements were categorized using k-means clustering (k = 5) (additional file 2: Fig. S2). A large portion of B1- and B2-containing peaks were present before LPS treatment (additional file 2: Fig. S1). It should be noted that the ChIP-seq data was for early LPS response in mice, therefore, it remains unknown whether the mouse SINEs are involved in late LPS response since the transcriptomic change was not evident at LPS 6 h in mice (additional file 2: Fig. S2).

To further investigate whether the human Alu subfamilies showed different histone modifications, we checked peaks from the AluJ (the oldest Alu), AluS, and AluY (the youngest) [37]. In both histone modifications, the youngest AluY was significantly under-represented relative to its genomic copy number (12% of total Alu) in all groups. Instead, the oldest AluJ (27% of total genomic Alu) was enriched (additional file 2: Fig. S3). Although the mechanistic rationale for AluY under-representation is unknown, we note that use of short sequencing reads and discarding multi-mapped reads may reduce mapping efficiency of AluY loci with high sequence similarity between them.

### Alu elements did not contribute to super-enhancer formation or organization under LPS stimulation

**Fig. 2:**
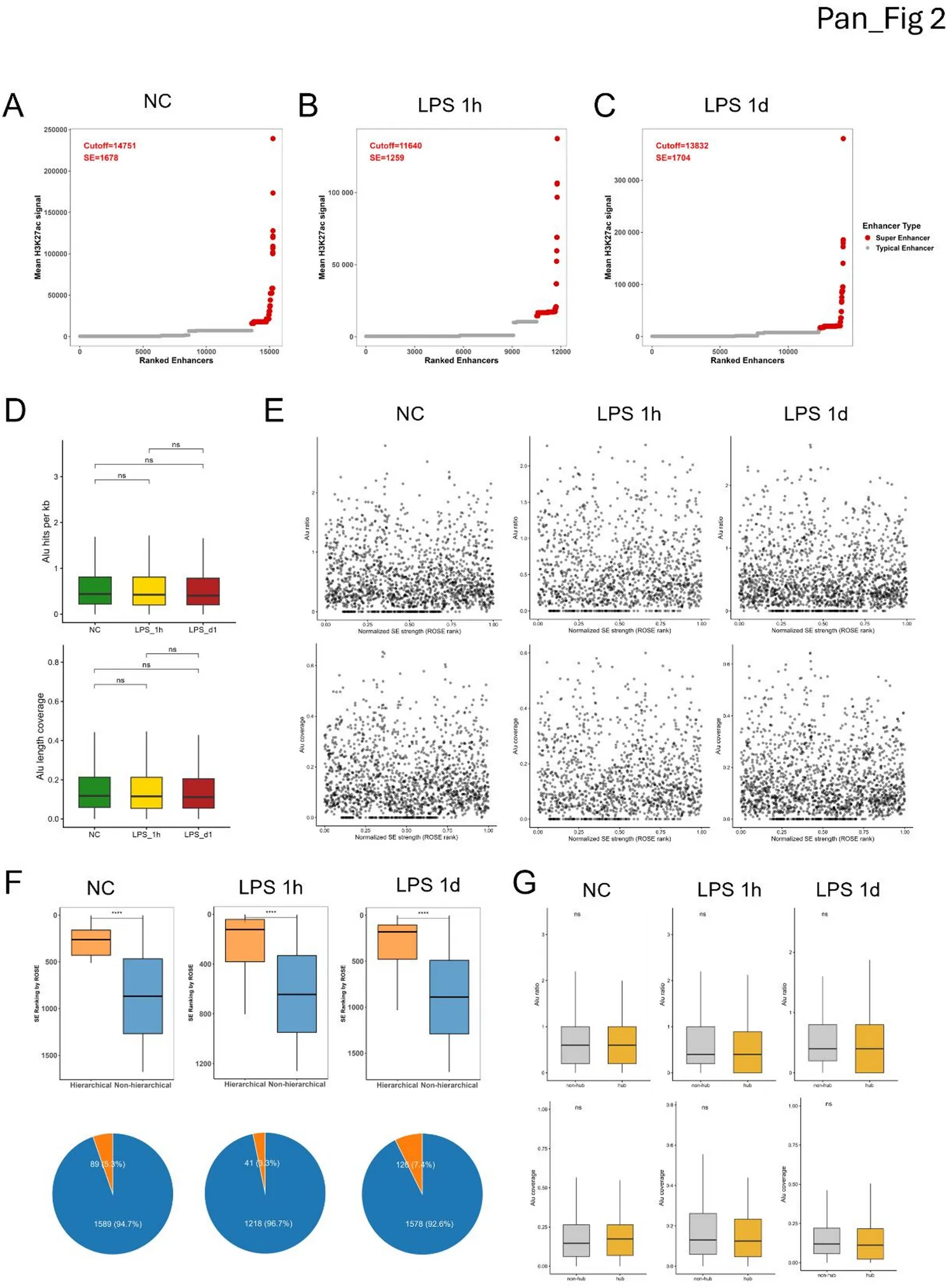
Alu elements do not contribute to super-enhancer formation or hierarchical structure. **A**–**C** Identification of super-enhancers (SEs) under three conditions (NC, LPS 1 h, and LPS 1d) based on H3K27ac signal ranking. The cutoff values and the number of SEs identified are shown on the left top corner. Red dots, SEs; gray dots, typical enhancers. **D** Statistics of the number of Alu loci per 1 kb [top] and the length coverage of Alu [bottom] in SE regions. **E** Scattered plots of normalized SE strength against Alu ratio (the number of Alu loci per 1 kb) [top] and Alu length coverage [bottom] in SE regions identified under each condition. The conditions are indicated on the top. **F** SE strength (ROSE rank) of hierarchical (orange) and non-hierarchical (blue) SEs identified under each condition. The numbers and length occupancy of hierarchical (orange) and non-hierarchical (blue) SEs are shown on the bottom. The conditions are indicated on the top. **G** Statistics of the number of Alu loci per 1 kb [top] and the length coverage of Alu [bottom] in non-hub (gray) and hub (orange) enhancers in hierarchical SEs regions identified under each condition. The conditions are indicated on the top

Genomic regions containing multiple enhancers with a higher level of H3K27ac are defined as super-enhancers (SE) [38], which could activate genes that mediate the monocyte/macrophage polarization and functions [39]. Therefore, we further analyzed super-enhancer (SE) organization. Super-enhancers were identified using the ROSE algorithm (See Methods), yielding 1,200 to 1,700 SEs in each condition with LPS 1d having the highest number of SEs (Fig. 2A and C). We quantified both the number of Alu elements (Alu hits) and the cumulative Alu length within SE regions (Fig. 2D). The old subfamilies, AluJ and AluS, were significantly enriched in SE regions, although the enrichment was not so high as that observed H3K27ac or H3K4me1 peaks (additional file 2: Fig. S4). No significant differences in Alu composition were observed between the NC and LPS-treated groups. In addition, correlation analyses revealed no association between SE signal strength and Alu abundance or length (Fig. 2E). Furthermore, Alu content did not differ between hierarchical SEs and non-hierarchical SEs [40, 41] (Fig. 2F), nor between hub and non-hub SEs (Fig. 2G). These results indicate that Alu elements do not influence SE formation, hierarchy, or signal intensity under LPS stimulation. These results indicate that Alu elements do not act as architectural drivers of enhancer strength or super-enhancer formation, but instead function as sequence-level regulatory modules that endow typical enhancers with LPS-responsive transcription factor binding capacity, thereby shaping the late-phase immune transcriptional program in human monocytes.

**Fig. 3:**
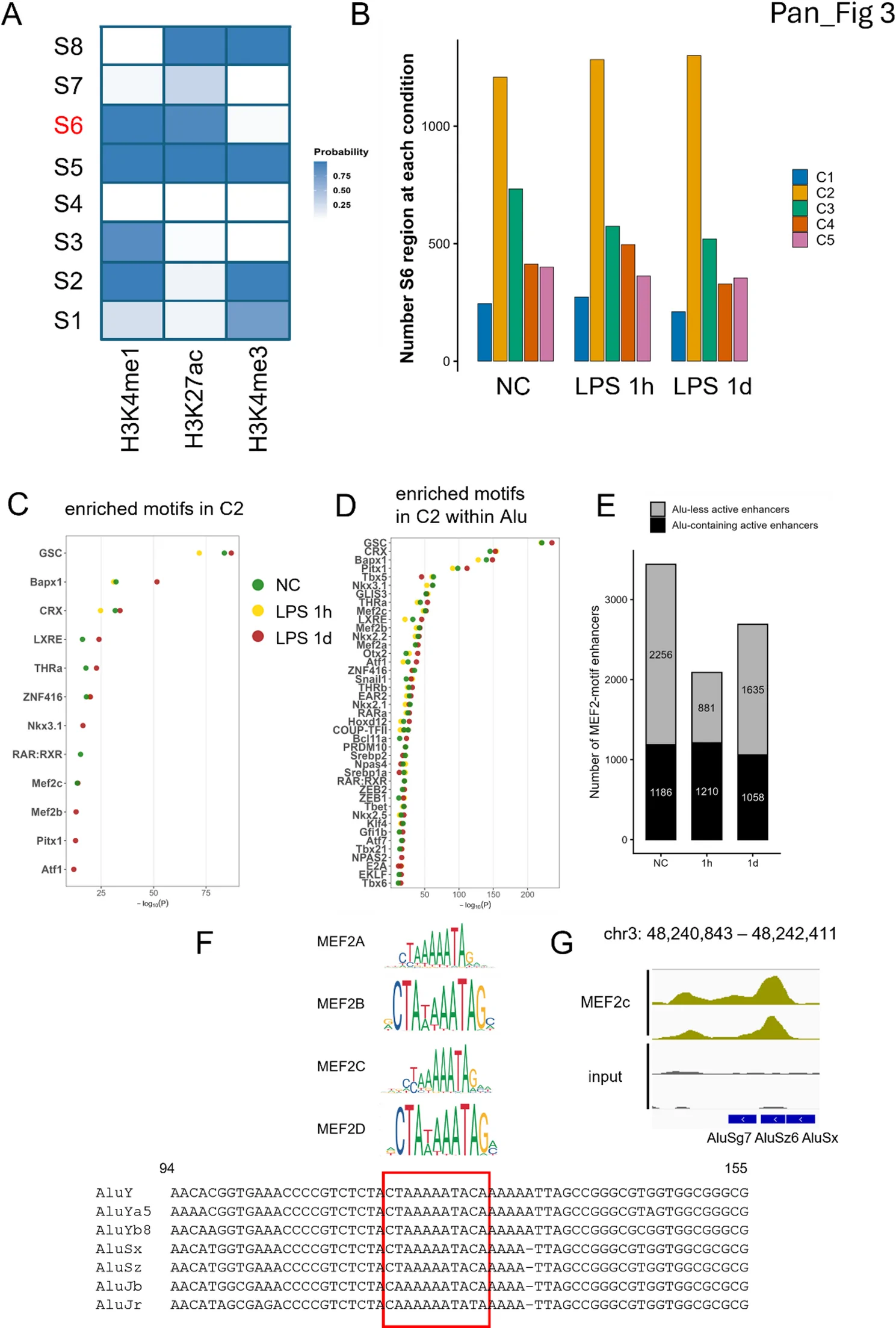
Alu elements provide multiple transcription factor binding sites. **A** ChromHMM segmentation based on H3K27ac, H3K4me1, and H3K4me3 signals. **B** Numbers of state S6 candidate enhancers across the five clusters in the three conditions. Clusters were defined by the H3K27ac signals. **C** HOMER motif enrichment analysis of S6 candidate enhancers in the C2 cluster. **D** HOMER motif enrichment analysis of the Alu sequences present in the S6 candidate enhancers in the C2 cluster. **E** Number of S6 candidate enhancers carrying MEF2 motifs under the three conditions. Candidate enhancers with and without Alu sequences are shown in black and gray, respectively. The conditions are indicated on the bottom. **F** Sequence alignment (positions 94 − 155 in AluY) of the consensus sequences of representative Alu subfamilies. The region matched to MEF2-family motif sequences is indicated by red rectangle. SequenceLogo representations of binding sequences for MEF2A to MEF2D are shown above. **G** Representative IGV tracks showing an Alu loci with MEF2C ChIP-seq signal in iPSC-derived microglia. The peak summit is located in the middle Alu copy (AluSz6).

We next examined the role of Alu at a finer regulatory scale. For identifying candidate enhancers, we applied ChromHMM using H3K4me1, H3K27ac and H3K4me3 ChIP-seq data (Fig. 3A). After assessing the reproducibility of ChromHMM (r values of 0.86-1.00) (additional file 1: Fig. S5), we defined regions with high H3K27ac and H3K4me1 and low H3K4me3 signals (state S6 in Fig. 3A) as enhancers. Approximately 30% of the Alu-contained H3K27ac peaks in C1 to C5 clusters were in the S6 state, and all subsequent analyses were restricted to this enhancer set (Fig. 3B).

We performed motif enrichment analysis on the Alu-containing candidate enhancers using HOMER, which revealed enrichment of binding motifs for Homeobox, zinc finger (ZF), and MADS-box families in cluster C2 (Fig. 3C), especially inside the Alu regions in cluster C2 (Fig. 3D). These observations suggest that Alu elements embedded within enhancers play a considerable role in the enhancer function by providing binding sites for these transcription factors (TFs). To test this hypothesis, TF-motif enrichment analysis was performed for each cluster, identifying some enriched motifs (Fig. 3C and additional file 2; Fig. S6). When sequences outside the Alu sequences were analyzed, no significant enrichment was found. Instead, the enrichment was confined to the Alu sequences (Fig. 3D and additional file 2; Fig. S6).

Notably, the majority of enriched motifs, including those from the MEF2 family (MEF2A, MEF2B, and MEF2C), were predominantly contributed by Alu-derived sequences. The MEF2 family has been identified as critical transcription factors for transcriptional regulation in the cellular response to LPS, including macrophage polarization, survival, and the selective modulation of specific LPS-induced gene subsets [42]. The MEF2 family proteins have preferred binding sequences (motifs), and mutations in these motifs abolish the binding and gene activating activity of MEF2 [43]. Therefore, we further examined whether MEF2-family motifs were present in Alu-containing enhancers as well as in all candidate enhancers. We used FIMO to scan for MEF2 family motifs in active enhancers across all three conditions (Fig. 3E). We found that approximately 1,100 Alu-containing enhancers harbored at least one MEF2 motif. In the NC condition, a lower proportion (34%) of candidate enhancers carried the MEF2 motifs. After LPS treatment, the proportion was increased (58% for LPS 1 h and 39% for LPS 1d). To further investigate the origin of these motifs, we analyzed Alu consensus sequences for MEF2 motif occurrence. Strikingly, the Alu consensus sequences contained a canonical MEF2 binding motif located around the 120th position (Fig. 3F), which is a part of a linker between the left and right arms and does not exist in the B1 or B2 sequences. This highlights a potential human-specific regulatory contribution of Alu elements; therefore, we re-analyzed a publicly available MEF2C ChIP-seq dataset generated from human iPSC-derived microglia, a myeloid cell type closely related to monocytes and macrophages, which identified a total of 60,809 MEF2C peaks. Among them, 8,116 peaks were located in Alu element (Fig. 3G), supporting that MEF2C indeed has ability to bind to Alu sequences, although significant enrichment was not seen.

### Stage-specific functional associations of Alu-containing enhancers during LPS stimulation

To assess the functional associations of Alu-containing enhancers, we assigned enhancer peaks to their putative target genes. To this end, we used published Hi-C and mRNA-seq data (see Methods). First, genes located close to an Alu-containing enhancer were identified by GREAT. Second, using the Hi-C data, genes located in the same topologically associating domains (TAD) as that enhancer were retained, given the fact that enhancer–promoter interactions are largely constrained within a TAD. Lastly, genes showing little expression in the given condition were filtered out (additional file 2: Fig. S7A). The distance between candidate enhancers and their presumed target genes was around or less than 10 kb with very few being over 100 kb (Additional file 2: Fig. S7B). In comparison to all genes showing TPM values of > 1 (used as background genes) (additional file 1: Table S4 and S5), these presumed target genes showed higher TPM levels (additional file 2; Fig. S8). Moreover, the fold changes in expression after the LPS 1d condition versus the NC condition were greater for the presumed target genes, which suggests that they were under the control of the candidate enhancers. The upstream enhancers of IL6, a well-known immune response gene [44] also overlapped with Alu-containing regions.

**Fig. 4:**
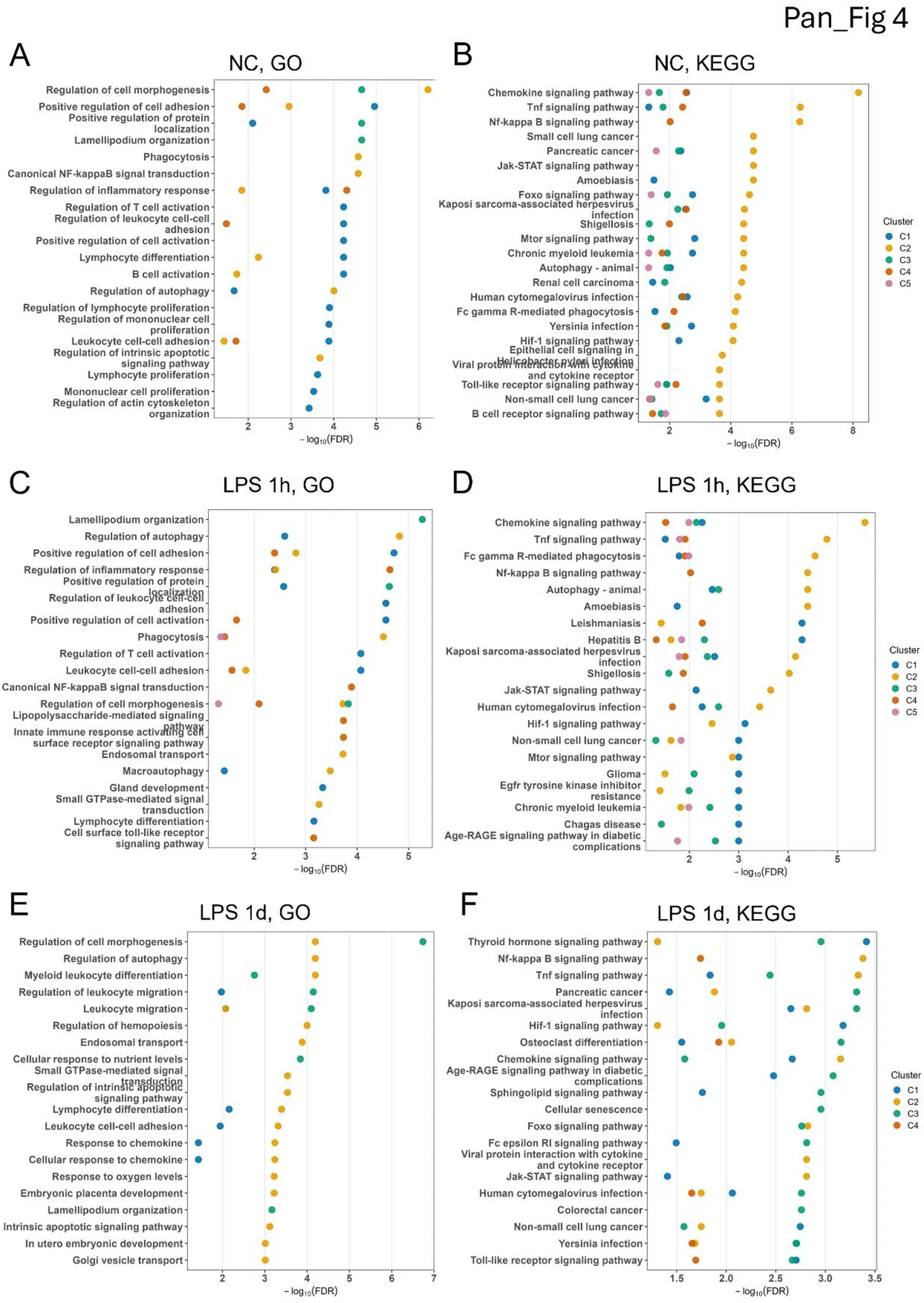
Alu-containing candidate enhancers exhibit dynamic functional changes upon LPS stimulation. Enriched Gene Ontology (GO) terms are shown for S6 candidate enhancers under the NC (**A**), LPS 1 h (**C**) and LPS 1d (**E**) conditions. Enriched KEGG pathways are shown for S6 candidate enhancers under the NC (**B**), LPS 1 h (**D**) and LPS 1d (**F**). In each condition, enrichment analysis was done for each cluster (C1 to C5 based on the H3K27ac signals)

Under each of NC, LPS 1 h and LPS 1d conditions, gene sets were defined separately for the five enhancer clusters (C1 to C5 in Fig. 3B) for Gene Ontology and KEGG pathway enrichment analyses (number of the genes in each gene set is provided in additional file 1: Table S6 and S7). The potential target genes of the enhancer clusters differed between conditions, disclosing condition-specific enhancer–gene associations with distinct functional enrichments. Genes associated with Alu-containing enhancers under NC conditions were enriched for cell morphology and adhesion and LPS-related signaling pathways, including NF-κB signaling in C1, C2 and/or C3 clusters (Fig. 4A and B). LPS 1 h-associated gene sets showed enrichment for pathways related to acute immune and inflammatory responses across multiple clusters (Fig. 4C and D). In contrast, LPS 1d-associated gene sets showed enrichment for diverse functions and pathways (Fig. 4E and F). Therefore, genes associated with Alu-containing enhancers and their functional enrichment profiles changed during the response to LPS.

## Discussion

In this study, we systematically investigated the involvement of Alu SINEs in the epigenomic response to LPS stimulation in human monocytes through integrative re-analysis of ChIP-seq, mRNA-seq, and Hi-C datasets. Our results reveal that Alu elements contribute to LPS-responsive enhancer activity by providing pre-existing regulatory potential, rather than by driving large-scale enhancer amplification.

LPS is a highly immunostimulatory molecule that humans encounter frequently in both external and internal environments [45]. Substantial interspecies differences in LPS responsiveness have been reported, despite the evolutionary conservation of key receptors involved in LPS recognition, including TLR4 and CD14. Previous studies have suggested that species-specific transcription factor binding landscapes and regulatory architectures may contribute to these differences.

TEs, particularly Alu elements, represent attractive candidates for mediating human-specific regulatory responses [16]. Alu elements are primate-specific, highly abundant, and have been increasingly recognized as contributors to regulatory innovation. In line with previous studies reporting stimulus-responsive roles of Alu elements in viral or bacterial contexts [11], we focused on Alu-associated chromatin regions in monocyte–macrophage lineage cells following LPS stimulation. We observed that Alu-containing regions exhibited pronounced and dynamic changes in enhancer-associated histone modifications, particularly H3K4me1 and H3K27ac, consistent with their roles in regulated enhancer activation rather than passive chromatin remodeling.

Importantly, we sought to exclude the possibility that the elevated H3K27ac signals observed at Alu sites simply reflected their inclusion within exceptionally strong enhancers. To this end, we examined SE organization and found that neither the abundance nor the cumulative length of Alu elements correlated with SE signal strength, hierarchy, or hub formation. Moreover, Alu composition within SEs remained stable across conditions. These observations argue against a model in which Alu-associated signals arise from super-enhancer–driven expansion of active regions. Instead, the results support that Alu elements function independently of higher-order enhancer structures.

At the sequence level, Alu elements exhibited a strong capacity to provide transcription factor binding sites. Motif analyses of the Alu containing enhancers revealed enrichment of binding sites of multiple families of transcription factors, including Homeobox, zinc finger, and MADS-box proteins, with the MEF2 family emerging as a prominent LPS-responsive factor [42, 46]. Notably, both HOMER and FIMO analyses demonstrated that the majority of these motifs were derived directly from Alu sequences rather than surrounding non-Alu regions. This finding suggests that Alu elements encode intrinsic regulatory information that has been amplified at various genomic places by retrotransposition and can subsequently be co-opted for the host immune response. This possibility is also supported by our results that NC-associated enhancers were enriched for pathways related to LPS sensing and immune preparedness, while LPS 1-hour stimulation preferentially activated acute immune response pathways, including NF-κB signaling. The LPS 1-day condition displayed more functionally dispersed enrichments, consistent with a transition toward post-stimulation adaptation or tolerance.

Together, these results indicate that Alu-containing enhancers do not act as drivers of sustained transcriptional amplification, but rather function as fine-scale regulatory modules that enable flexible, stage-specific transcriptional responses during LPS stimulation. By providing pre-positioned transcription factor binding platforms, Alu elements may facilitate rapid immune activation while allowing subsequent functional diversification as stimulation persists. This model highlights a previously underappreciated role of Alu elements in shaping the temporal epigenomic organization during innate immune responses in humans. It should be noted that the ~ 15% overlap of H3K27ac or H3K4me1 peaks with Alu sequences (Fig. 1A and B) was less than expected values (~ 27% by random permutation tests) (additional file 2: Table S8). Giving that the AluJ/AluS/AluY consensus sequences carry a MEF2 binding motif (Fig. 3F), each copy likely had an LPS-responsive activation potential when they were generated by retrotransposition. However, the presence of a large number (up to a million) of LPS-responsive regions with no functional role may be a disadvantage for the host to appropriately control the transcriptomic change after bacterial infection. Therefore, Alu loci that did not activate genes important for the LPS response may have undergone natural selection for not being activated under LPS exposure by mutations and/or stable repressive chromatin states. Functionally important, a limited number of Alu loci may have been selected so that they have kept the activity of the LPS-responsive enhancer.

A limitation of the present study is the lack of direct perturbation experiments targeting specific Alu elements. Future studies employing targeted deletion or epigenome editing will be required to establish causal relationships between individual Alu insertions and immune regulatory outcomes. In addition, recently published T2T genomes [47] may increase the completeness of identification of SINE-derived enhancers, as they carry about 1% higher number of SINE copies than the reference genomes used here (hg38 and mm39). To avoid the multi-mapping artifacts, we only used uniquely mapped reads for rigorous identification of ChIP-seq peaks. However, we put a caveat that discarding multi-mapped reads underestimates the contribution of young TEs such as AluY (additional file 1: Table S9). Use of long read sequencing should be a next challenge for the epigenome and transcriptome studies focusing on evolutionarily young TEs.

## Supplementary Information

Below is the link to the electronic supplementary material.

Supplementary Material 1.

Supplementary Material 2.

## Data Availability

The public dataset could be obtained from GSE91009, GSE85245, GSE306993, GSE201376, GSE201353, GSE281922.
