# Supplementary material for: Human Alu loci gain enhancer-type epigenetic modifications in monocytes during response to bacterial LPS
Source: Epigenetics Chromatin. 2026 Jul 10;19:40. doi: 10.1186/s13072-026-00686-x (PMC13584426; doi:10.1186/s13072-026-00686-x)
Supplement: Supplementary file 1 [file 13072_2026_686_MOESM2_ESM.pdf]

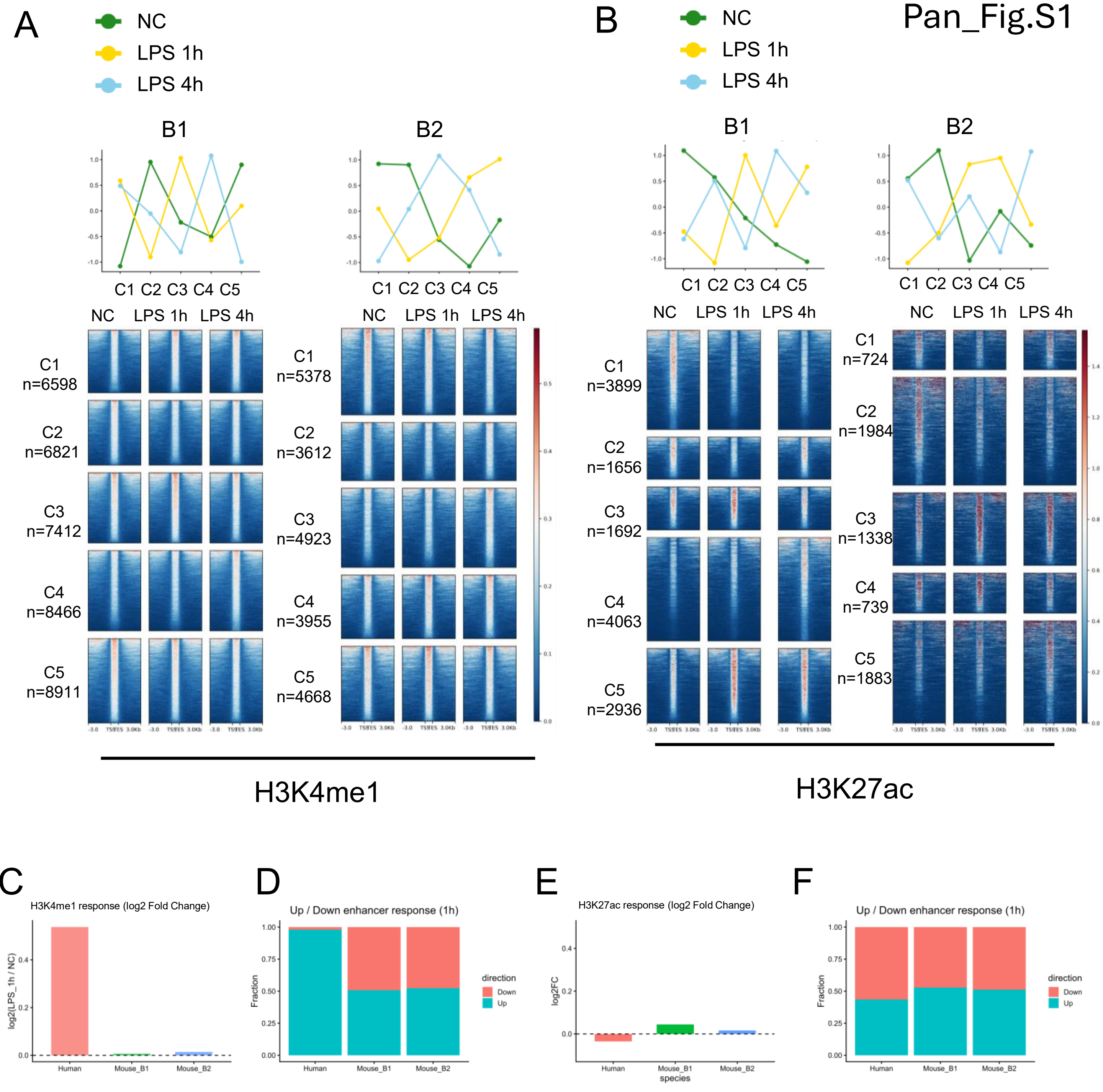

Legend is in the next page.

Fig. S1. B1/B2-containing peaks display similar clustering patterns to human Alu but show reduced signal dynamics.

(A) [top] Line graphs showing average H3K4me1 signal intensities of B1- and B2-containing ChIP-seq peaks in the C1 to C5 clusters under the NC (green), LPS 1h (yellow), and LPS 1d (red) conditions. [bottom] heat maps showing H3K4me1 signal intensities in individual peaks. B1-containing and B2-containing peaks are respectively categorized into C1 to C5 clusters. The number of peaks in each category is indicated. (B) [top] Line graphs showing average H3K27ac signal intensities of B1- and B2-containing ChIP-seq peaks in the C1 to C5 clusters under the NC (green), LPS 1h (yellow), and LPS 1d (red) conditions. [bottom] heat maps showing H3K27ac signal intensities in individual peaks. B1-containing and B2-containing peaks are respectively categorized into C1 to C5 clusters. The number of peaks in each category is indicated. (C) Average log2 fold changes in the H3K4me1 level by 1 h of LPS treatment at human Alu-, mouse B1-, and mouse B2-containing peaks. (D) Fraction of the human Alu-, mouse B1-, and mouse B2-containing H3K4me1 peaks with increased and decreased signals. (E) Average log2 fold changes in the H3K27ac level by 1 h of LPS treatment at human Alu-, mouse B1-, and mouse B2-containing peaks. (F) Fraction of the human Alu-, mouse B1-, and mouse B2-containing H3K27ac peaks with increased and decreased signals.

Pan_Fig.S2

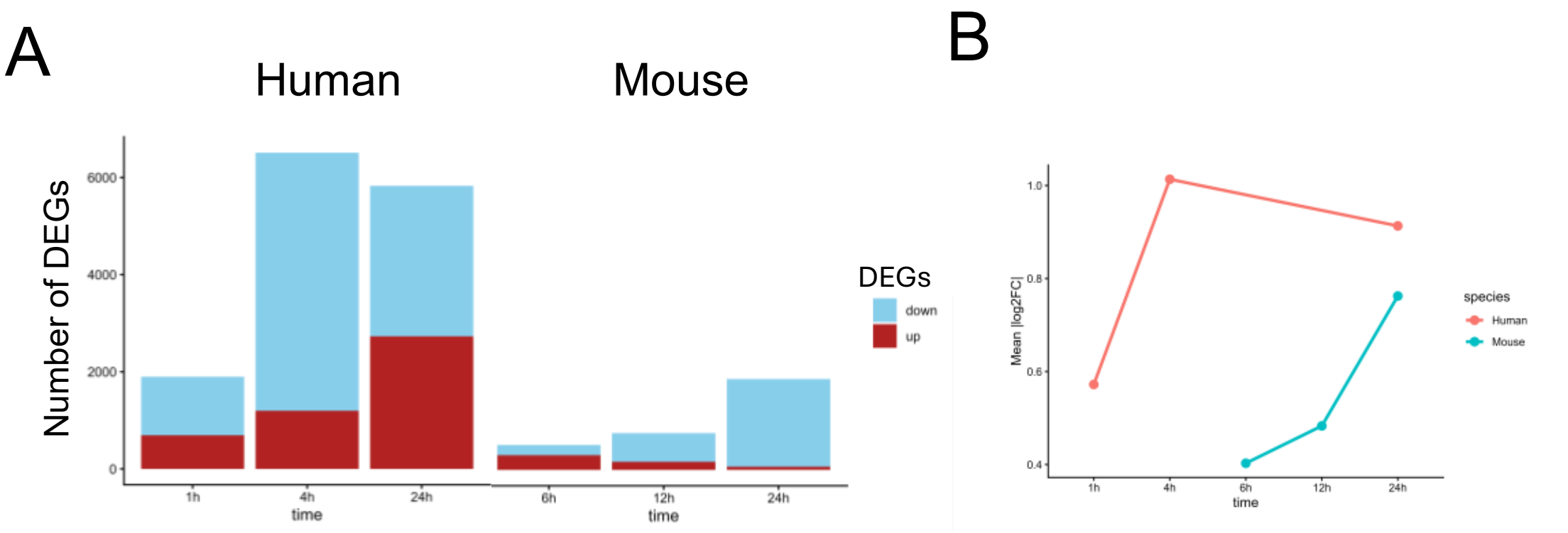

Fig. S2. Human CD14+ showed stronger response to LPS than mouse RAW264.7.
(A) The number of differentially expressed changed genes in human and mouse at each time point after LPS treatment. (B) Averages of gene expression changes (absolute log2 fold-change values) in human and mouse along the time after LPS treatment.

Pan_Fig.S3

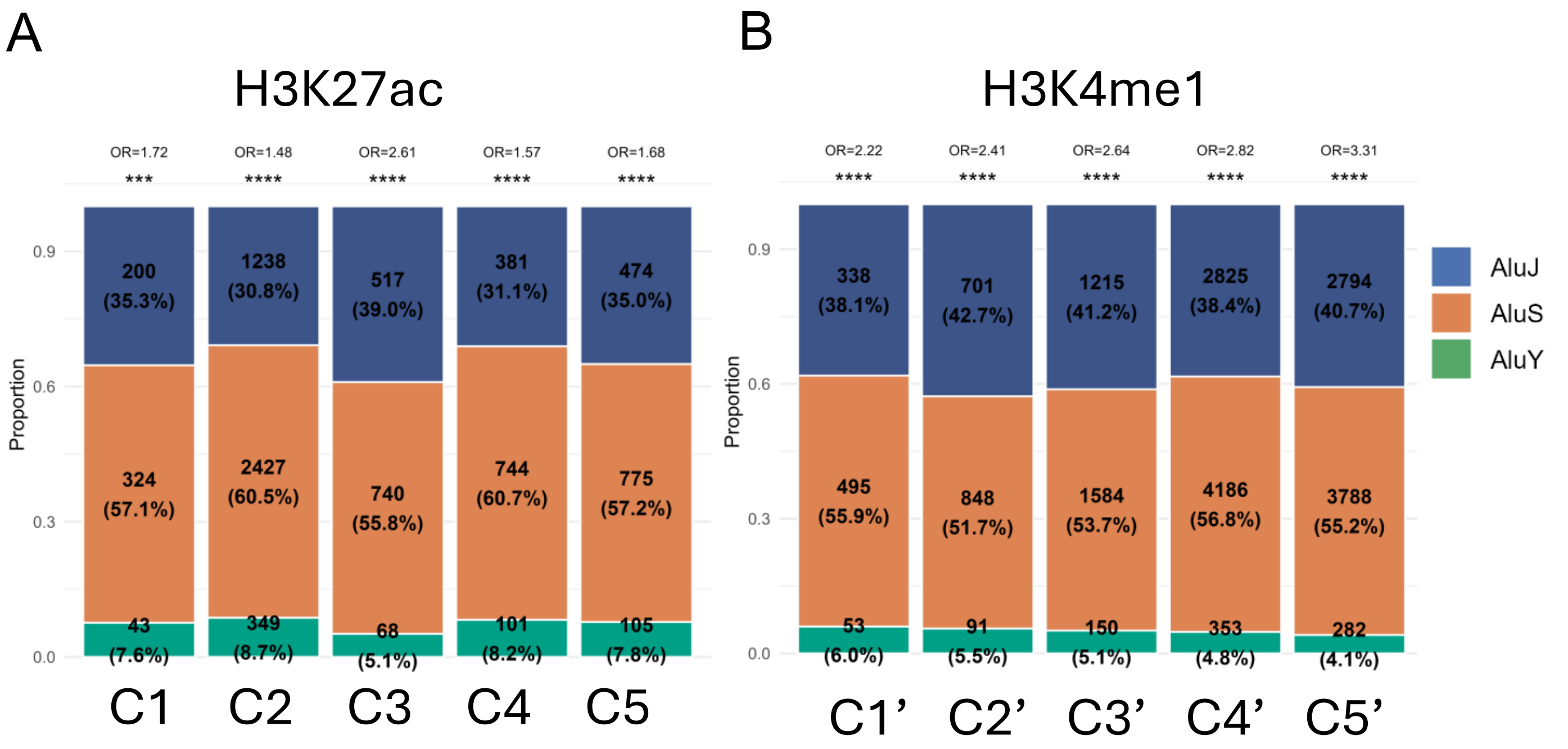

Genomic copies: AluJ = 27.3%, AluS = 60.3%, AluY = 12.4%.

Fig. S3. The enhancer-type histone modification preferred old Alus.
The proportion of the Alu subfamilies in H3K27ac (A) and H3K4me1 (B) peaks in each cluster. For statistical tests, We regarded the AluJ and AluS as old Alu, and AluY as young Alu. Then, we constructed a 2 × 2 contingency table based on the number of old versus young Alu elements in peak regions and in the genome. Fisher’s exact test was applied using fisher.test() in R to calculate odd ratios (OR) and p values. *** for $p < 0.001$ and **** for $p < 0.0001$.

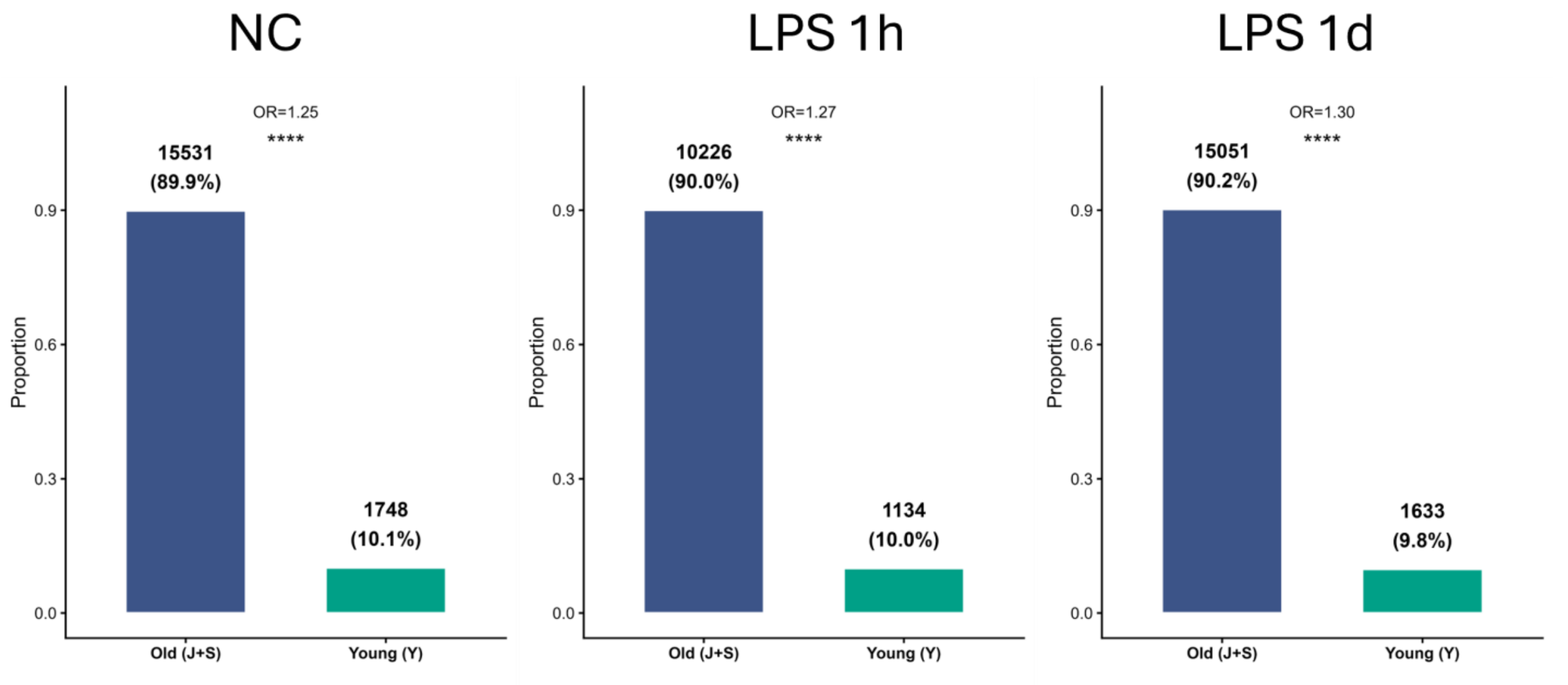

Fig. S4. Enrichment of old Alu loci in super-enhancers than young Alu loci.
For statistical tests, We regarded the AluJ and AluS as old Alu, and AluY as young Alu. Then, we constructed a 2 × 2 contingency table based on the number of old versus young Alu elements in peak regions and in the genome. Fisher's exact test was applied using fisher.test() in R to calculate odd ratios (OR) and p values. *** for $p < 0.001$ and **** for $p < 0.0001$.

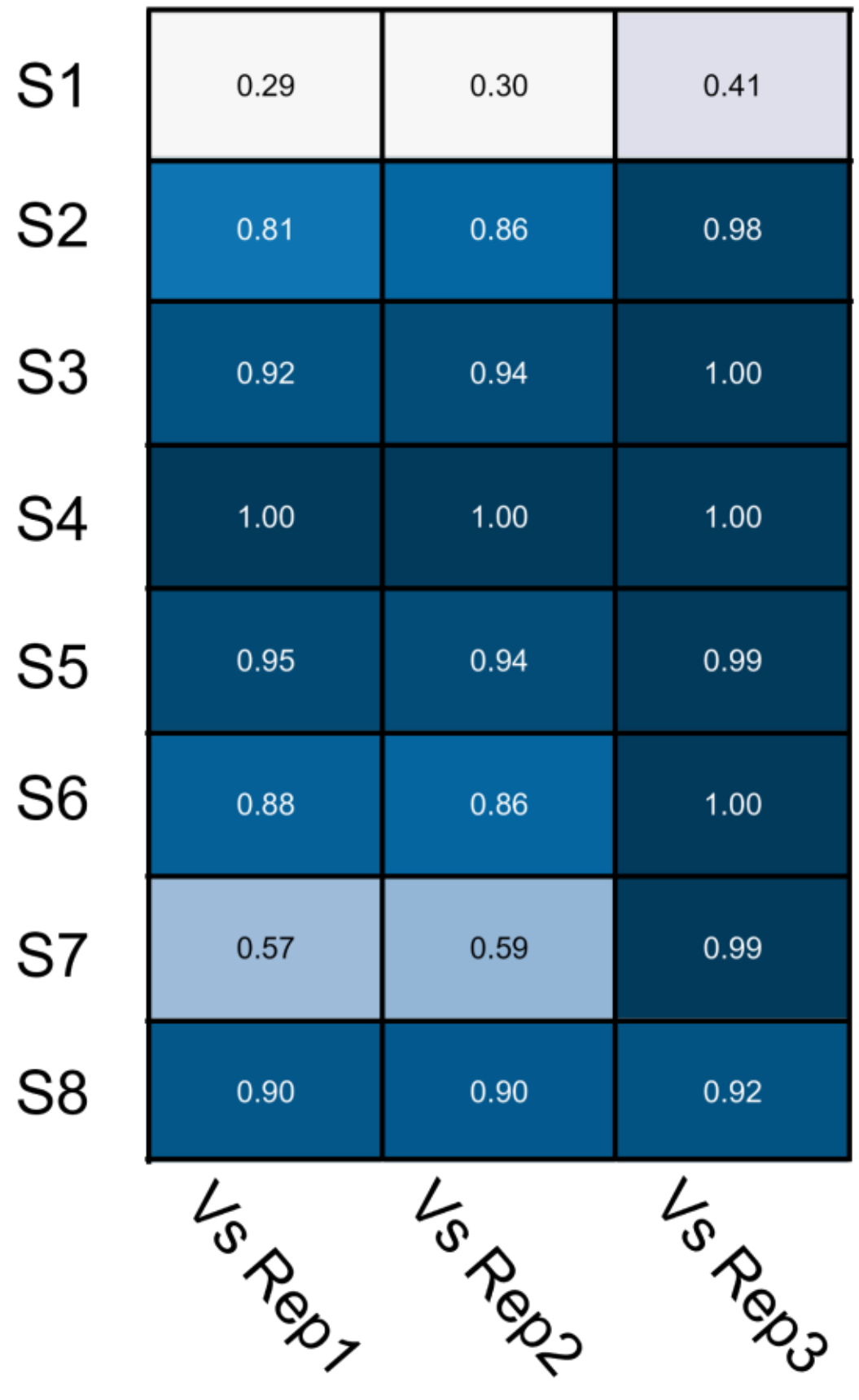

Fig. S5. The chromHMM results showed high consistency.
Three other runs of ChromHMM script were conducted with the parameter -s 123, -s 456, or -s 789 as technical replicates. To check their consistency, SAGAconf_parser.py and SAGAconf.py were used with the default settings. The GW average r_values in r_values_report.txt are shown for the original one versus rep1, rep2, and rep3, respectively.

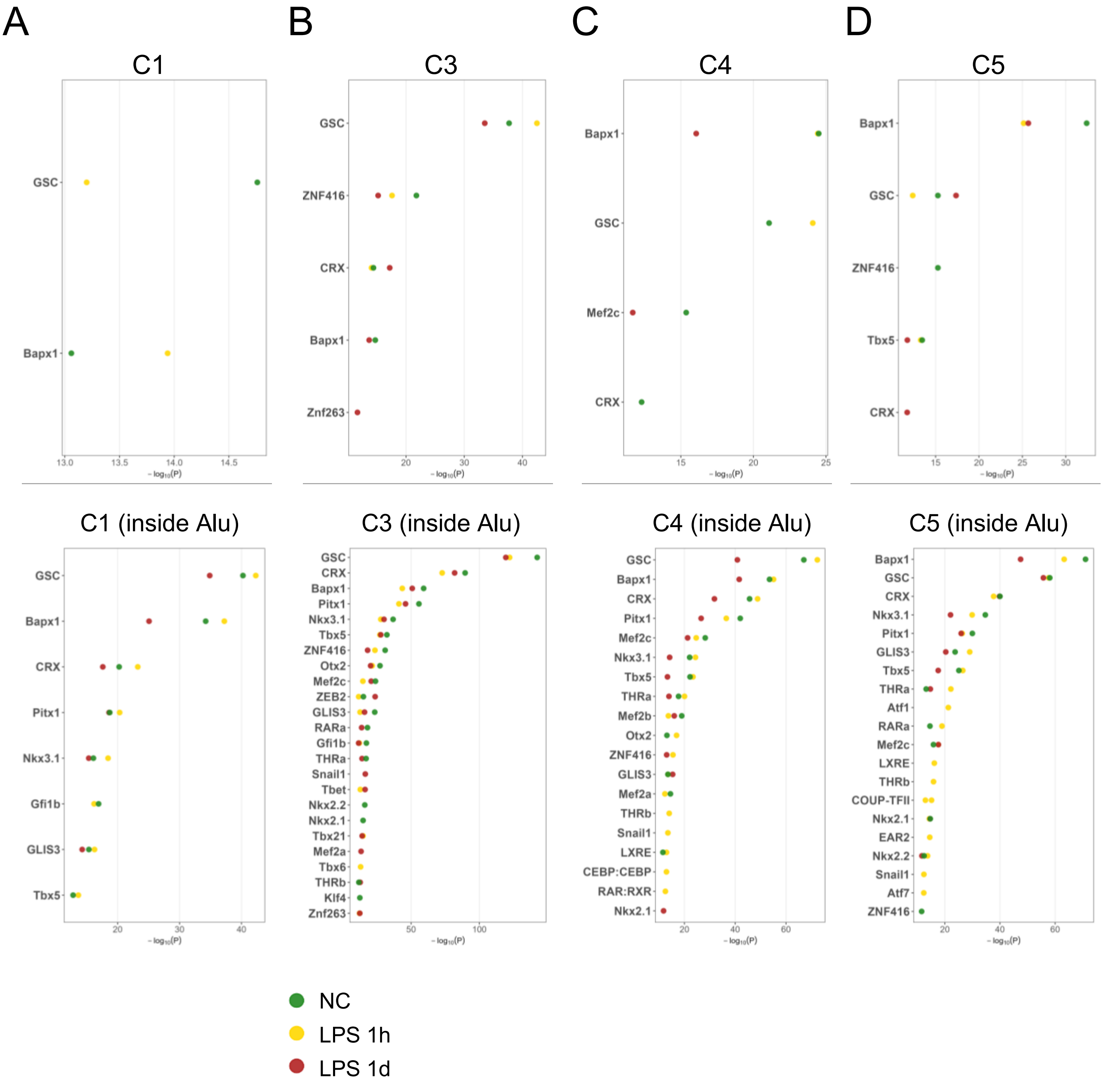

Fig. S6. Alu elements provide multiple transcription factor binding sites.
HOMER motif analysis of enhancer candidates (in the S6 state) of the C1 (A), C3 (B), C4 (C), and C5 (D) clusters using the whole sequences (top) and the sequences of the embedded Alu. The conditions (NC, LPS 1h, and LPS 1d) in which the enhancer-type histone modifications were detected are indicated by colors (green, yellow, and red, respectively).

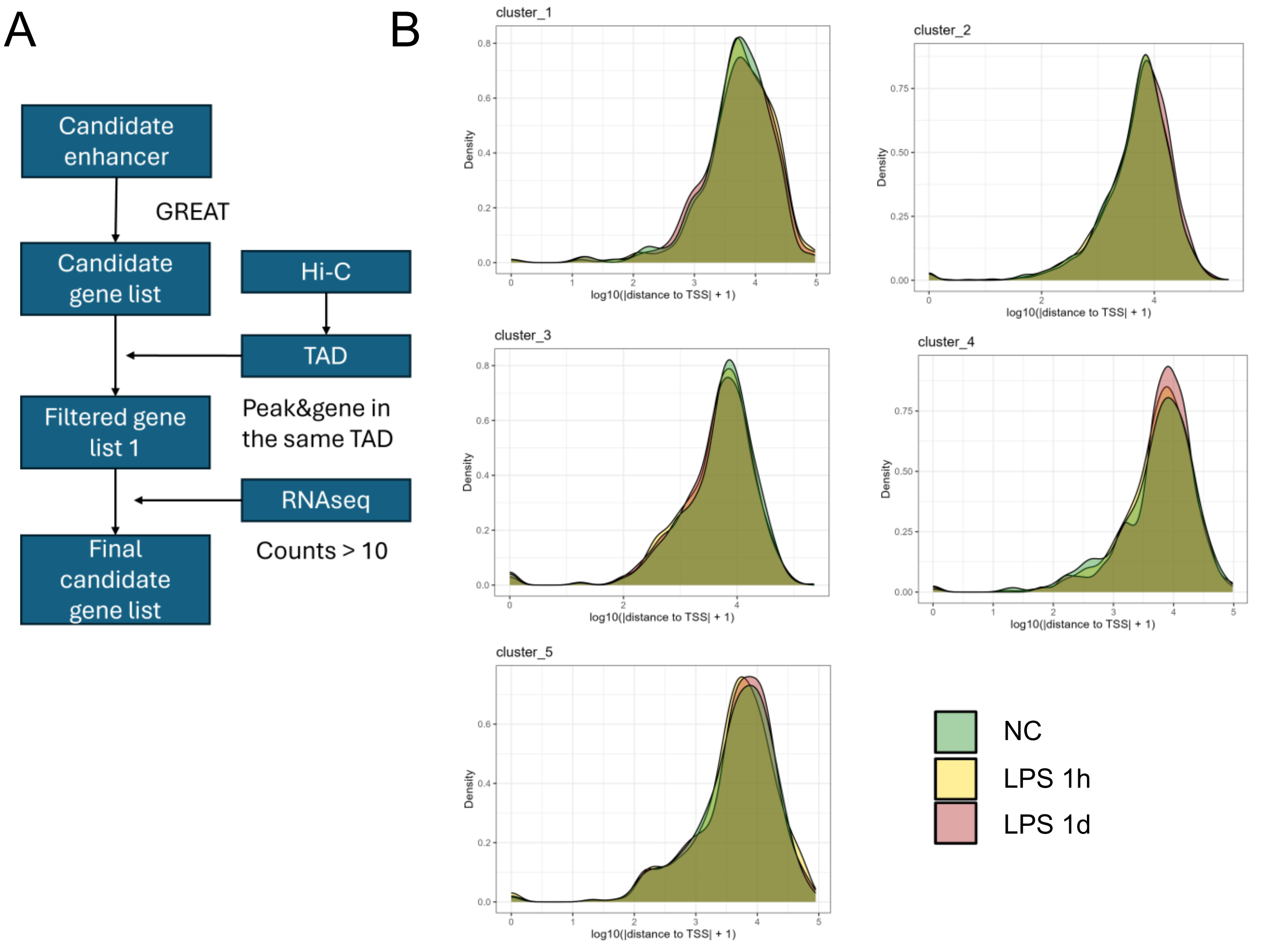

Fig. S7. Mapping of enhancer candidates to associated genes.
(A) Workflow for assigning enhancer candidates to genes. (B)The distribution of the distance between the enhancers and their associated genes identified in C1 to C5 clusters.

Pan_Fig.S8

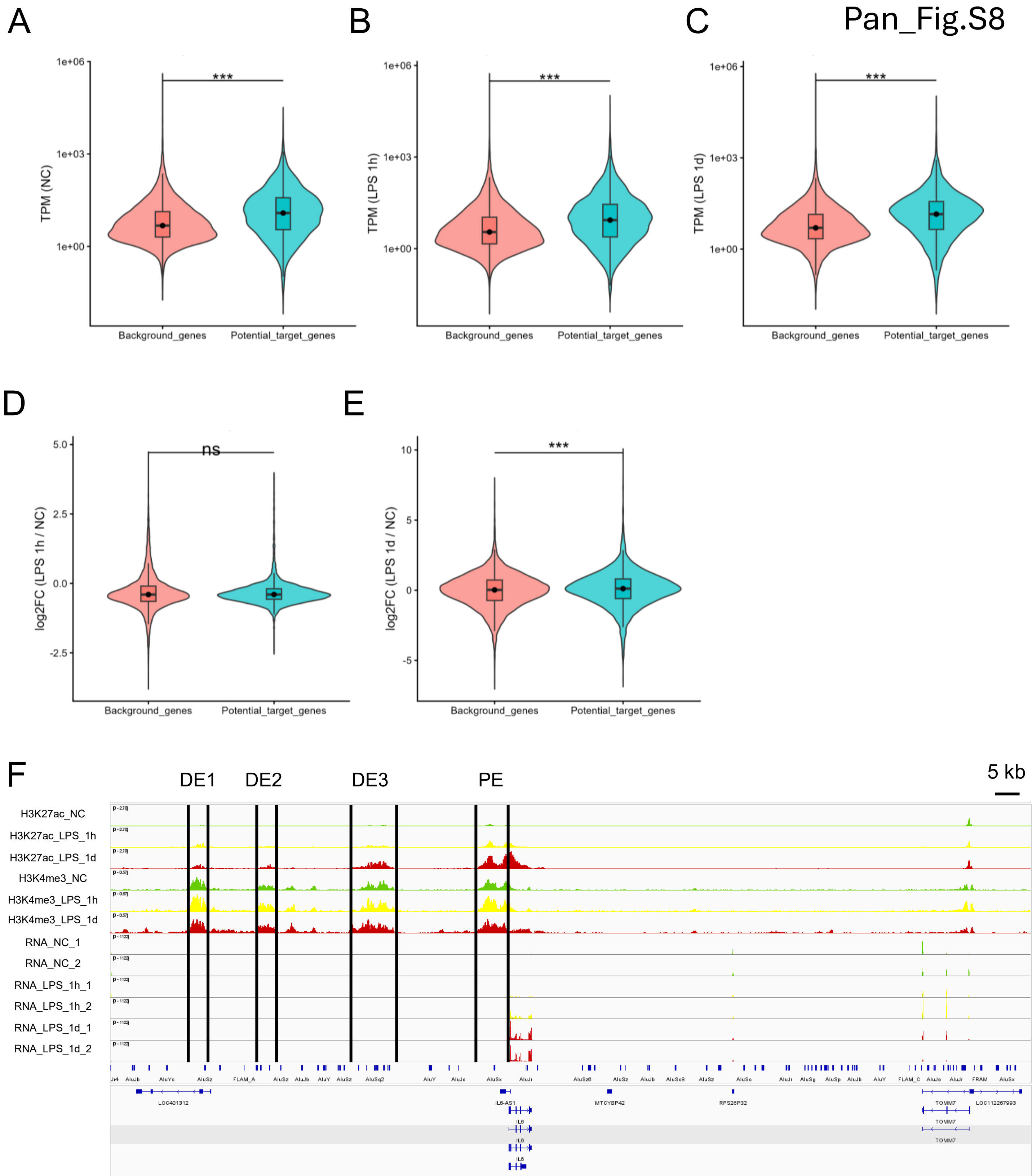

Fig. S8. Presumed target genes showed higher expression level compared to the background (A, B, and C) The expression level of background genes and the presumed target genes in NC, LPS 1h, and LPS 1d (D and E) The fold change compared with NC in presumed target genes and background. (F) Representative IGV tracks showing H3K27ac and H3K4me1 signals around the IL6. DE: distal enhancer, PE: proximal enhancer.
